# Outcomes of Lobar and Sublobar Resection for Clinical Stage I Lung Neuroendocrine Tumors: An ENETS Center of Excellence Experience †

**DOI:** 10.3390/jcm14227927

**Published:** 2025-11-08

**Authors:** Ranin Hojerat, Islam Idais, Gal Aviel, Anat Bel-Ange, Simona Grozinsky-Glasberg, Simona Ben-Haim, Benjamin Nisman, Ofra Maimon, Karine Atlan, Oz M. Shapira, Amit Korach, Uzi Izhar, Guy Pines, Ori Wald

**Affiliations:** 1 Department of Cardiothoracic Surgery, Hadassah Medical Center, Faculty of Medicine, Hebrew University of Jerusalem, Jerusalem 91120, Israel; 2 Neuroendocrine Tumor Unit, ENETS Centre of Excellence, Division of Internal Medicine, Hadassah Medical Center and Faculty of Medicine, Hebrew University of Jerusalem, Jerusalem 91120, Israel; 3 Department of Medical Biophysics and Nuclear Medicine, Hadassah Medical Center and Faculty of Medicine, Hebrew University of Jerusalem, Jerusalem 91120, Israel; 4 Institute of Nuclear Medicine, University College London Hospitals NHS Foundation Trust, 235 Euston Road, London NW1 2BU, UK; 5 Department of Oncology, Hadassah Medical Center and Faculty of Medicine, Hebrew University of Jerusalem, Jerusalem 91120, Israel; 6 Department of Pathology, Hadassah Medical Center and Faculty of Medicine, Hebrew University of Jerusalem, Jerusalem 91120, Israel; 7 Department of Thoracic Surgery, Kaplan Medical Center and Faculty of Medicine, Hebrew University of Jerusalem, Jerusalem 91120, Israel

**Keywords:** lung neuroendocrine tumors, pulmonary carcinoids, lobar resection, sublobar resection, pro-gastrin-releasing peptide

## Abstract

**Objectives:** Lung neuroendocrine tumors (LNETs) are rare, comprising 1–2% of lung cancers. This study aimed to compare overall survival (OS) and recurrence-free survival (RFS) after lobar resection versus sublobar resection for LNETs and to identify factors associated with prognosis and resection extent. **Methods:** We retrospectively analyzed patients with clinical stage I (T ≤ 4 cm, N0M0) typical or atypical carcinoid who underwent curative resection at Hadassah Medical Center and Kaplan medical Center between 2010 and 2024. **Results:** Seventy patients (mean age 56.8 ± 16 years; 63% female) were included. Lobar resection was performed in 40 (57%) and sublobar resection in 30 (43%; 15 segmentectomies, 15 wedge resections). Pathology revealed 50 typical carcinoid (71.43%) and 20 atypical carcinoid (28.57%). Final pathological stage was I in 57 patients (81.42%), II in 9 (12.86%), and III in 4 (5.71%), reflecting surgical upstaging in 13 patients (18.57%), all due to nodal involvement. Atypical carcinoid was associated with worse RFS, nodal upstaging, and adjuvant therapy (all *p* < 0.01). Patients undergoing sublobar resection were older, had higher comorbidity scores, more frequently presented with peripheral tumors, and underwent less frequent lymph node assessment (all *p* < 0.01). At a median follow-up of 3.8 years for OS and 2.0 years for RFS, survival rates were 95.7% for both. Neither OS, RFS, nor postoperative normalization of plasma pro-gastrin-releasing peptide (ProGRPp) levels differed significantly between lobar resection and sublobar resection (*p* = 0.94, *p* = 0.42, and *p* = 0.205, respectively). **Conclusions:** Sublobar resection may represent an acceptable surgical option for selected patients with clinical stage I LNETs, particularly for peripheral tumors ≤ 2 cm in older or comorbid patients. The high rate of nodal upstaging underscores the need for lymph node assessment, irrespective of resection extent.

## 1. Introduction

Lung neuroendocrine tumors (LNETs), also referred to as pulmonary carcinoids (PCs), are rare neoplasms with an annual incidence of approximately 0.2–2 cases per 100,000 individuals in both the United States and Europe, accounting for 1–2% of all primary lung cancers and 20–25% of all neuroendocrine tumors [1]. In recent years, both the incidence and prevalence of LNETs have shown an upward trend, likely due to increased awareness, advancements in diagnostic techniques and a possible true rise in disease occurrence [2].

According to the 2021 World Health Organization (WHO) Classification of Thoracic Tumors, LNETs fall under the broader group of lung neuroendocrine neoplasms, which also includes high-grade neuroendocrine carcinomas (LNECs) [3]. LNETs comprise well-differentiated typical carcinoid (TC)—defined by <2 mitoses per 2 mm^2^ and absence of necrosis—accounting for 85–90% of cases, and atypical carcinoid (AC)—defined by 2–10 mitoses per 2 mm^2^ with focal necrosis—accounting for 10–15%. In contrast, LNECs comprise poorly differentiated small cell lung carcinoma (SCLC) and large cell neuroendocrine carcinoma (LCNEC), which exhibit > 10 mitoses per 2 mm^2^ and extensive necrosis [3]. Diffuse idiopathic pulmonary neuroendocrine cell hyperplasia (DIPNECH)—a rare proliferation of neuroendocrine cells within the peripheral airway mucosa—is considered a precursor lesion that may progress to LNETs [4].

Although this histologic classification implies a continuum from low- to high-grade neuroendocrine neoplasms, molecular evidence indicates that LNETs and LNECs arise from distinct oncogenic pathways, representing biologically separate entities. LNETs have a relatively low burden of chromosomal alterations and rarely harbor smoking-associated TP53 or RB1 mutations—hallmarks of high-grade LNECs [5]. Accordingly, LNETs carry a favorable prognosis, with 10-year disease-specific survival rates of 96% and 85% for stage I and II TC and 88% and 75% for stage I and II AC, respectively [6].

Given their low mutational burden and indolent growth—with tumor doubling times of approximately 2.7 years for TC and 0.9 years for AC [7]—surgical resection remains the mainstay of treatment for localized LNETs, over 80% of which present at early stages (TNM I–II) [6]. Although lobectomy has long been considered the standard approach, advances in imaging and the increasing use of cross-sectional studies have led to earlier detection of smaller, often incidental, localized tumors, prompting growing interest in sublobar resection —including both anatomical segmentectomy and wedge resection—for selected early-stage cases. This approach is further supported by evidence from two recent randomized trials in early-stage non–small cell lung cancer (NSCLC)—a biologically more aggressive disease yet serving as the basis for current staging and surgical management guidelines for LNETs—where sublobar resection achieved oncologic outcomes comparable to lobectomy [8,9].

Current clinical guidelines regarding the optimal extent of surgical resection for localized LNETs remain variable [10]. The European Society for Medical Oncology (ESMO, 2021) [6] and the European Neuroendocrine Tumor Society (ENETS, 2015) [11] favor lobectomy over segmentectomy and note that wedge resection is associated with increased recurrence—particularly in N-positive typical carcinoid (TC) or atypical carcinoid (AC). In contrast, the National Comprehensive Cancer Network (NCCN, 2021) [12] and the North American Neuroendocrine Tumor Society (NANETS, 2020) [13] endorse both lobectomy and segmentectomy as appropriate options. Furthermore, in 2020, the Commonwealth Neuroendocrine Tumour Research Collaboration (CommNETs) and NANETS jointly updated the 2015 ENETS consensus, suggesting that sublobar resection may be acceptable for peripheral TC < 2 cm, provided complete (R0) resection is achievable [14].

Historically, lymph node (LN) dissection was often omitted owing to the perceived indolent nature of LNETs; however, recent studies have demonstrated a non-negligible incidence of nodal involvement and a prognostic impact of LN assessment [15]. Nevertheless, the role and extent of LN evaluation in LNETs remain heterogeneous. ESMO 2021 [6], ENETS 2015 [11], and CommNETs/NANETS 2020 [14] advocate systematic nodal dissection, with removal of at least six nodal stations (three hilar and three mediastinal, including the subcarinal station), consistent with the European Society of Thoracic Surgeons (ESTS) recommendations for NSCLC. In contrast, NANETS 2020 [13] and NCCN 2021 [12] consider hilar or mediastinal nodal sampling acceptable.

This variability in both surgical and nodal management underscores the lack of consensus regarding optimal treatment strategies. The primary objective of this study was to compare overall survival (OS) and recurrence-free survival (RFS) in patients with clinical stage I LNETs undergoing lobar resection (LR) versus sublobar resection (SLR). Secondary objectives were to identify prognostic factors for adverse outcomes and determinants influencing the extent of resection. We hypothesized that OS would be comparable between LR and SLR, but omission of lymph node assessment would be associated with inferior survival.

## 2. Materials and Methods

We conducted a retrospective cohort study of patients with LNETs who underwent curative-intent resection between 2010 and 2024 at Hadassah Medical Center and Kaplan Medical Center.

Eligible patients were adults (≥18 years) with histologically confirmed typical carcinoid (TC) or atypical carcinoid (AC) and clinical stage I disease, defined as tumor size ≤4 cm without lymph node involvement or distant metastasis, according to the 8th edition of the American Joint Committee on Cancer (AJCC) Staging Manual. Patients with histologically mixed tumors (e.g., combined LNETs and non-small cell lung carcinoma), metastatic or unresectable disease at presentation, or incomplete medical records were excluded. Clinical, operative, pathological, and follow-up data were retrieved from electronic medical records. All patients underwent either lobectomy or sublobar resection (including wedge resection or segmentectomy).

Preoperative evaluation included clinical assessment, serum hematology and biochemistry, pulmonary function testing, and chest radiography, together with measurement of chromogranin A, progastrin-releasing peptide (ProGRP), and, when functional disease was suspected, additional hormonal assays. Preoperative tissue diagnosis was feasible in 53% of patients, obtained via bronchoscopy in 97% and CT-guided transthoracic biopsy for peripheral tumors in 3%. Staging included contrast-enhanced chest CT and either fluorodeoxyglucose (FDG) positron emission tomography (PET) or 68Ga-DOTATATE PET; 87% of patients underwent at least one PET modality, and 25% underwent both. All cases were reviewed at a multidisciplinary tumor board to assess surgical resectability and physical operability, defined by adequate performance status and preserved pulmonary, cardiac, renal, and hepatic function. The surgical approach—open thoracotomy versus minimally invasive video-assisted (VATS) or robot-assisted (RATS) thoracoscopic resection—was at the surgeon’s discretion, with minimally invasive techniques preferred for peripheral lesions and thoracotomy reserved for central or larger tumors.

Overall survival (OS) was calculated from the date of surgery to death from any cause, and recurrence-free survival (RFS) from the date of surgery to the first documented recurrence, with patients alive or recurrence-free censored at the date of last follow-up.

The study was approved by the Institutional Review Board of Hadassah Medical Center (IRB No. 0170-19-HMO, 31 July 2024) in accordance with the principles of the Declaration of Helsinki.

### Statistical Analysis

Continuous variables were summarized as means with standard deviations when normally distributed and as medians with interquartile ranges when not. Comparisons between two independent groups were performed using the independent two-sample *t*-test when assumptions of normality and homogeneity of variance were met, and the Mann–Whitney U test otherwise. Categorical variables were expressed as frequencies and percentages and compared using the Chi-square test when expected cell counts were ≥5 and Fisher’s exact test when expected counts were <5.

Survival outcomes were analyzed using the Kaplan–Meier method, and differences between groups were compared with the log-rank test. All statistical tests were two-sided, and *p*-values < 0.05 were considered statistically significant. All statistical analyses were performed using IBM SPSS Statistics for Windows, Version 30.0 (IBM Corp., Armonk, NY, USA).

## 3. Results

### 3.1. Patient Characteristics

According to the selection criteria, 70 patients were included in the final analysis; 62 underwent surgery at Hadassah Medical Center and 8 at Kaplan Medical Center. Clinical and pathological characteristics are summarized in Table 1 and Table 2 according to tumor grade (typical vs. atypical carcinoid) and extent of resection (lobar vs. sublobar), respectively. The mean age at surgery was 56.76 ± 16 years, and the majority of patients were female (*n* = 44, 63%). Pathology revealed 50 typical carcinoids (71.43%) and 20 atypical carcinoids (28.57%). As expected, atypical carcinoids exhibited more aggressive features, including larger tumor size, higher Ki-67 index, lymphovascular invasion, perineural invasion, and invasion of adjacent structures (all *p* < 0.05). Additionally, atypical carcinoid was associated with higher rates of nodal upstaging (*p* < 0.01) and receipt of adjuvant therapy (*p* < 0.001).

### 3.2. Surgical Treatment

Lobar resection (LR) was performed in 40 patients (57.14%), and sublobar resection (SLR) in 30 (42.86%), including 15 segmentectomies (21.43%) and 15 wedge resections (21.43%). Compared with LR, patients undergoing SLR were older, had higher Charlson Comorbidity Index scores [16], and were more likely to have peripheral tumors (all *p* ≤ 0.01). Additionally, patients in SLR group were more frequently treated using minimally invasive approaches—video-assisted thoracoscopic surgery (VATS) or robot-assisted thoracoscopic surgery (RATS)—(*p* ≤ 0.01) and had a shorter length of hospital stay (LOS) (median [IQR], 3 [2,3] vs. 5 [4,5,6] days; *p* ≤ 0.01; Table 2).

### 3.3. Lymph Node Assessment

SLR was associated with less extensive lymph node assessment, including both hilar and mediastinal stations (all *p* < 0.01; Table 3). Final pathological stage was I in 57 patients (81.4%), II in 9 (12.9%), and III in 4 (5.7%), reflecting surgical upstaging in 13 patients (18.6%), all attributable to nodal involvement (Table 2). Not surprisingly, SLR was associated with lower rates of nodal upstaging compared with LR (*p* = 0.03; Table 2).

### 3.4. Survival Analysis

**Figure 1:**
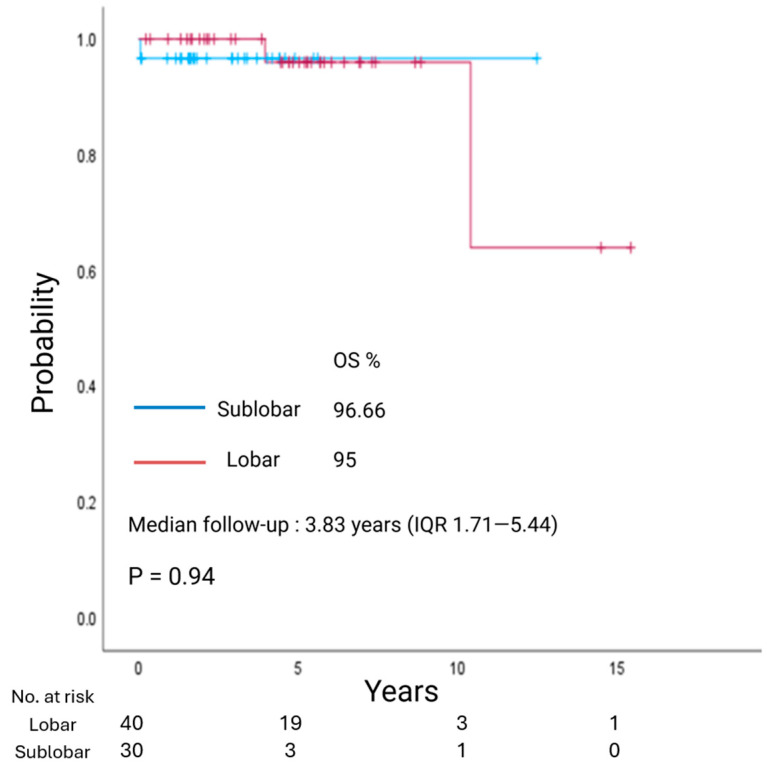
Kaplan–Meier estimate of overall survival (OS) in patients with clinical stage I lung neuroendocrine tumors undergoing lobar resection or sublobar resection.

**Figure 2:**
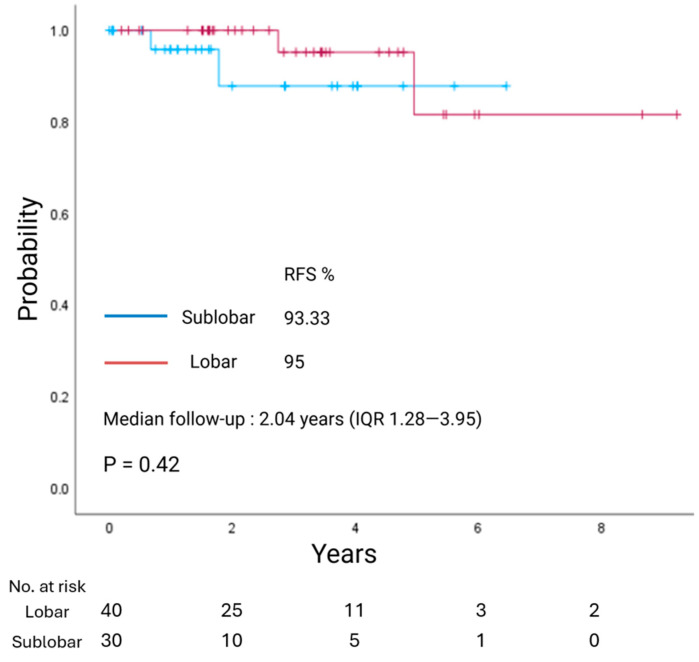
Kaplan–Meier estimate of recurrence-free survival (RFS) in patients with clinical stage I lung neuroendocrine tumors undergoing lobar resection or sublobar resection.

With a median follow-up of 3.83 years (IQR 1.71–5.44), overall survival (OS) did not differ between LR and SLR groups (*p* = 0.94; Figure 1). Similarly, with a median follow-up of 2.04 years (IQR 1.28–3.95), recurrence-free survival (RFS) showed no significant difference between the two groups (*p* = 0.42; Figure 2).

### 3.5. Post-Operative Plasma Progastrin-Releasing Peptide (ProGRPp)

**Figure 3:**
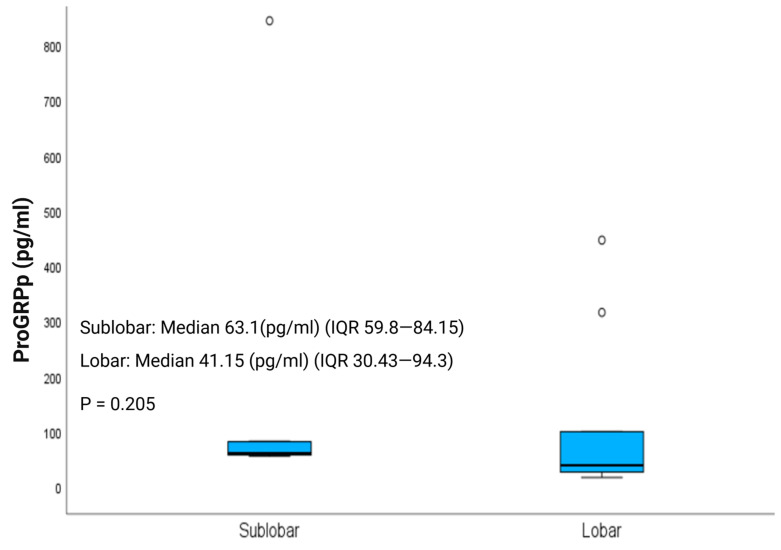
Postoperative ProGRPp (Plasma Progastrin-Releasing Peptide) levels in patients with clinical stage I lung neuroendocrine tumors undergoing lobar resection (LR) or sublobar resection (SLR).

Postoperative ProGRPp levels, a biomarker recently proposed as a measure of disease burden in LNETs [17], normalized to a similar extent in the LR and SLR groups, with median values of 41.15 pg/mL (IQR, 30.43–94.3) and 63.1 pg/mL (IQR, 59.8–84.15), respectively (*p* = 0.205; Figure 3). Notably, ProGRPp (normal range, 0–65 pg/mL) remained elevated in patients with pathologically confirmed diffuse idiopathic pulmonary neuroendocrine cell hyperplasia (DIPNECH), despite resection of the dominant nodule, reflecting persistent disease burden.

### 3.6. Prognostic Factors

Univariate survival analyses using the Kaplan–Meier method with log-rank testing identified atypical carcinoid histology, presence of necrosis, elevated Ki-67 index, visceral pleural invasion, administration of adjuvant somatostatin analog (SSA) therapy, and pathological lymph node upstaging as predictors of worse RFS (all *p* < 0.05). In contrast, functional tumors (ACTH-secreting), high Ki-67 index, and visceral pleural invasion were associated with worse OS (all *p* < 0.05; Table 4).

## 4. Discussion

Lung neuroendocrine tumors (LNETs) are rare, accounting for 1–2% of all primary lung cancers [1], and their optimal management remains controversial [9].

In this retrospective cohort study, we compared outcomes of lobar resection (LR) and sublobar resection (SLR) in patients with clinical stage I LNETs. We found no significant differences in overall survival (OS) or recurrence-free survival (RFS) between the two approaches. To our knowledge, this is the first report to demonstrate that plasma pro-gastrin–releasing peptide (ProGRPp)—an emerging biomarker of disease burden and post-treatment surveillance [16]—normalizes to a similar extent after both LR and SLR, supporting the possible oncologic adequacy of sublobar approaches in selected patients.

This study represents the largest LNETs experience reported from Israel and the Middle East, adding geographic and demographic diversity to the body of evidence on LNETs management.

Given that approximately 80% of our cohort had N0 tumors ≤ 2 cm and that R0 resection was achieved in SLR at rates comparable to LR, our findings, in accordance with previous studies [18,19,20,21,22,23,24,25,26,27,28,29], suggest that SLR may represent an acceptable surgical alternative in selected patients with clinical stage IA–IB LNETs, particularly in older or comorbid patients unfit for more extensive surgery.

Importantly, consistent with prior reports [15,19,25,29,30,31], our findings underscore the necessity of lymph node assessment regardless of resection type. Nodal involvement is not uncommon in LNETs, occurring in 10–15% of TC and 30–60% of AC [10,29,32,33]. In our series, nodal upstaging was significantly associated with worse RFS and, as demonstrated in other studies [19,25,30,31,34], correlated with inferior OS, indicating that lymph node status is a more critical determinant of long-term outcomes than the extent of pulmonary resection.

Given the non-negligible rate of nodal involvement, comprehensive lymph node assessment should not be omitted when selecting SLR, as inadequate LN evaluation may compromise staging accuracy and prognostic validity. SLR should be reserved for cases with optimized preoperative evaluation, including FDG- and/or ^68^Ga-DOTATATE PET-CT and tissue diagnosis when feasible, which may aid in identifying the emerging subset of LNETs with elevated proliferation—characterized by carcinoid morphology but mitotic counts exceeding 10 per 2 mm^2^ and/or more extensive necrosis [35].Our study has several limitations. The retrospective design carries inherent risks of confounding, selection bias, and incomplete data. The cohorts were not fully balanced for baseline characteristics, with differences in age, comorbidity burden, and tumor location potentially influencing outcomes. The modest sample size and limited number of events, reflecting the rarity and indolent course of LNETs, further constrained statistical power and precluded advanced bias-mitigation methods. Finally, the relatively short follow-up may underestimate late recurrences, which are well recognized in this disease.

## 5. Conclusions

In conclusion, our findings indicate that sublobar resection may be an acceptable surgical option for selected patients with clinical stage IA–IB lung neuroendocrine tumors (LNETs), particularly those with peripheral lesions, advanced age, or significant comorbidities unsuitable for extensive resection. This approach should be employed cautiously, following comprehensive preoperative staging and adequate intraoperative lymph node assessment, given the established prognostic significance of nodal involvement.

Additional studies are warranted to validate these findings and 35better define optimal patient selection and surgical strategies through large, multi-institutional prospective cohorts, complemented by systematic reviews and meta-analyses to strengthen the evidence base for surgical management of LNETs.

## Figures and Tables

**Table 1:** Baseline characteristics stratified by tumor grade (Typical vs. Atypical Carcinoid).

Parameter	Total (*n* = 70)	Typical (*n* = 50)	Atypical (*n* = 20)	*p*-Value
Demographics and Clinical Features				
Sex (F/M, % F)	44/26 (63%)	30/20 (60%)	14/6 (70%)	0.43
Age (years), mean ± SD	56.76 ± 16	57.50 ± 15.80	55 ± 17.10	0.51
CCI, median (IQR)	2 (1–4)	2 (0.75–4.00)	2.50 (1.00–3.75)	0.80
Preoperative FEV1 (% predicted), median (IQR)	92 (80–99)	92 (80–99)	88 (81.50–101)	0.86
Smoking *	22 (35.48%)	14 (28%)	8 (40%)	0.33
Previous malignancy	19 (32.25%)	14 (32%)	5 (28%)	0.80
Tumor location (C/P, % C)	25/45 (35.71%)	20/30 (40%)	5/15 (25%)	0.94
Functional tumor **	3 (4.29%)	2 (4%)	1 (5%)	1.00
Minimally invasive approach ***	33 (47.14%)	22 (44%)	11 (55%)	0.40
LOS (days), median (IQR)	4 (3–5)	4 (3–5)	5 (3–6)	0.78
30-day mortality	1 (1.43%)	1 (2%)	0	1.00
Adjuvant therapy (SSA)	16 (22.86%)	5 (10%)	11 (55%)	**<0.001**
**Nuclear Imaging**				
FDG SUVmax, median (IQR)	2.96 (2–4.40)	2.78 (2–3.49)	4.35 (2.62–5.42)	0.08
68Ga-DOTATATE SUVmax, median (IQR)	9.10 (1.80–34.06)	12.20 (2.38–30.10)	2.00 (0–44.81)	0.59
**Type of Surgery**				0.07
Lobectomy	40 (57.14%)	25 (50%)	15 (75%)	
Sublobar resection	30 (42.86%)	25 (50%)	5 (25%)	
Segmentectomy	15 (21.43%)	13 (26%)	2 (10%)	
Wedge resection	15 (21.43%)	12 (24%)	3 (15%)	
**Pathological Parameters**				
Mitotic index (per 2 mm^2^), mean ± SD	1.47 ± 1.95	0.67 ± 0.60	3.47 ± 2.66	**<0.001**
Necrosis	15 (22.73%)	0	15 (75%)	**<0.001**
Ki-67 index (%), mean ± SD	4.62 ± 4.80	2.36 ± 1.72	9.94 ± 5.54	**<0.001**
DIPNECH	17 (24.29%)	12 (24%)	5 (26%)	1.00
Lymphovascular invasion	14 (21.21%)	6 (13%)	8 (40%)	**0.02**
Perineural invasion	8 (12.70%)	3 (7%)	5 (28%)	**0.04**
Visceral pleural invasion	3 (4.55%)	1 (2%)	2 (10%)	0.19
Direct invasion of adjacent structures	7 (10.61%)	2 (4%)	5 (25%)	**0.02**
Margins (R0/R1, %R0)	63/5 (92.65%)	45/3 (93.75%)	18/2 (90%)	0.62
**Clinical Staging**				
**T category**				**0.04**
T1a (≤1 cm)	15 (21.43%)	12 (24%)	3 (15%)	
T1b (>1 but ≤2 cm)	41 (58.57%)	32 (64%)	9 (45%)	
T1c (>2 but ≤3 cm)	11 (15.71%)	4 (8%)	7 (35%)	
T2a (>3 but ≤4 cm)	3 (4.29%)	2 (4%)	1 (5%)	
T2b–T4 (>4 cm)	0	0	0	
**N category**				
N0	69 (98.57%)	49 (98%)	20 (100%)	
N1–N3	0	0	0	
Nx	1 (1.43%)	1 (2%)	0	
**M category**				
M0	70 (100%)	50 (100%)	20 (100%)	
M1	0	0	0	
Clinical Stage ****				
Stage I	70 (100%)	50 (100%)	20 (100%)	
Stage II–IV	0	0	0	
**Pathological Staging**				
**T category**				**<0.01**
T1a (≤1 cm)	15 (21.43%)	13 (26%)	2 (10%)	
T1b (>1 but ≤2 cm)	34 (48.57%)	27 (54%)	7 (35%)	
T1c (>2 but ≤3 cm)	13 (18.57%)	4 (8%)	9 (45%)	
T2a (>3 but ≤4 cm)	8 (11.43%)	6 (12%)	2 (10%)	
T2b–T4 (>4 cm)	0	0	0	
**N category**				**<0.01**
N0	50 (71.43%)	40 (80%)	10 (50%)	
N1	11 (15.71%)	3 (6%)	8 (40%)	
N2	2 (2.86%)	1 (2%)	1 (5%)	
N3	0	0	0	
Nx	7 (10%)	6 (12%)	1 (5%)	
Pathological Stage ****				**0.01**
Stage I	57 (81.43%)	45 (90%)	12 (60%)	
Stage II	9 (12.86%)	4 (8%)	5 (25%)	
Stage III	4 (5.71%)	1 (2%)	3 (15%)	
Stage IV	0	0	0	
**LN Assessment and Upstaging**				
LN assessment *****	63 (90%)	45 (90%)	18 (90%)	1.00
Pathologic upstaging	13 (18.57%)	5 (10%)	8 (40%)	**<0.01**
LN upstaging	13 (18.57%)	5 (10%)	8 (40%)	**<0.01**

* Functional tumors: all functional cases were ACTH–producing. ** Smoking: includes current and former smokers. *** Minimally invasive approach: includes VATS and RATS. **** Best available staging—when Nx, N0 was assumed. ***** Including lymph node dissection, sampling or unknown type of lymph node assessment. **Abbreviations:** ACTH = adrenocorticotropic hormone; CCI = Charlson Comorbidity Index; DIPNECH = diffuse idiopathic pulmonary neuroendocrine cell hyperplasia; FDG = 18F-fluorodeoxyglucose; 68Ga-DOTATATE = gallium-68-DOTA-Tyr3-Thre8-octreotide; FEV1 = forced expiratory volume in one second; F/M = female/male; IQR = interquartile range; LN = lymph node; LOS = length of hospital stay; RATS = robot-assisted thoracic surgery; R0/R1 = negative/positive resection margin; SD = standard deviation; SSA = somatostatin analog; SUVmax = maximum standardized uptake value; VATS = video-assisted thoracoscopic surgery; Tumor location (C/P, % C) = central/peripheral, % central.

**Table 2:** Baseline characteristics stratified by type of surgical resection (LR vs. SLR).

Parameter	Total (*n* = 70)	Lobar (*n* = 40)	Sublobar (*n* = 30)	*p*-Value
Demographics and Clinical Features				
Sex (F/M, % F)	44/26 (63%)	21/19 (52.50%)	23/7 (76.67%)	0.07
Age (years), mean ± SD	56.76 ± 16	50.58 ± 17.20	64.50 ± 9.72	**<0.001**
CCI, median (IQR)	2 (1–4)	1.50 (0–4)	3 (2–4.75)	**<0.01**
Preoperative FEV1 (% predicted), median (IQR)	92 (80–99)	92 (79.50–97)	92 (85–99)	0.81
Smoking *	22 (35.48%)	14 (38.89%)	8 (30.77%)	0.70
Previous malignancy	19 (32.25%)	8 (22.22%)	11 (42.30%)	0.16
Tumor location (C/P, % C)	25/45 (35.71%)	21/19 (52.50%)	4/26 (13.33%)	**<0.001**
Functional tumor **	3 (4.29%)	1 (2.50%)	2 (6.67%)	0.57
Minimally invasive approach ***	33 (47.14%)	13 (32.50%)	20 (66.67%)	**0.01**
LOS (days), median (IQR)	4 (3–5)	5 (4–6)	3 (2–3)	**<0.01**
30-day mortality	1 (1.43%)	0	1 (3.33%)	0.43
Adjuvant therapy (SSA)	16 (22.86%)	10 (25%)	6 (20%)	0.78
**Nuclear Imaging**				
FDG SUVmax, median (IQR)	2.96 (2–4.40)	3.10 (2.50–5)	2.80 (2–4.25)	0.46
68Ga-DOTATATE SUVmax, median (IQR)	9.10 (1.80–34.06)	24.75 ± 30.10	13.45 ± 15.47	0.31
**Pathological Parameters**				
Typical/atypical (% typical)	50/20 (71.43%)	25/15 (62.50%)	25/5 (83.33%)	0.10
Mitotic index (per 2 mm^2^), mean ± SD	1.47 ± 1.95	1.58 ± 1.92	1.33 ± 2.01	0.94
Necrosis	15 (22.73%)	11 (28.94%)	4 (14.29%)	0.24
Ki-67 index (%), mean ± SD	4.62 ± 4.80	5.17 ± 5.19	3.86 ± 4.17	0.17
DIPNECH	17 (24.29%)	6 (15%)	11 (36.67%)	0.20
Lymphovascular invasion	14 (21.21%)	9 (23.68%)	5 (17.86%)	0.76
Perineural invasion	8 (12.70%)	7 (20%)	1 (3.57%)	0.13
Visceral pleural invasion	3 (4.55%)	2 (5.41%)	1 (3.45%)	1.00
Direct invasion of adjacent structures	7 (10.61%)	6 (16.22%)	1 (3.45%)	0.23
Margins (R0/R1, %R0)	63/5 (92.65%)	35/4 (89.74%)	28/1 (96.55%)	0.62
**Clinical Staging**				
**T category**				0.16
T1a (≤1 cm)	15 (21.43%)	6 (15%)	9 (30%)	
T1b (>1 but ≤2 cm)	41 (58.57%)	23 (57.50%)	18 (60%)	
T1c (>2 but ≤3 cm)	11 (15.71%)	8 (20%)	3 (10%)	
T2a (>3 but ≤4 cm)	3 (4.29%)	3 (7.50%)	0	
T2b–T4 (>4 cm)	0	0	0	
**N category**				
N0	69 (98.57%)	40 (100%)	29 (96.67%)	
N1–N3	0	0	0	
Nx	1 (1.43%)	0	1 (3.33%)	
**M category**				
M0	70 (100%)	40 (100%)	30 (100%)	
M1	0	0	0	
**Clinical Stage** ****				
Stage I	70 (100%)	40 (100%)	30 (100%)	
Stage II–IV	0	0	0	
**Pathological Staging**				
**T category**				0.16
T1a (≤1 cm)	15 (21.43%)	6 (15%)	9 (30%)	
T1b (>1 but ≤2 cm)	34 (48.57%)	18 (45%)	16 (53.33%)	
T1c (>2 but ≤3 cm)	13 (18.57%)	10 (25%)	3 (10%)	
T2a (>3 but ≤4 cm)	8 (11.43%)	6 (15%)	2 (6.67%)	
T2b–T4 (>4 cm)	0	0	0	
**N category**				**<0.01**
N0	50 (71.43%)	29 (72.50%)	21 (70%)	
N1	11 (15.71%)	10 (25%)	1 (3.33%)	
N2	2 (2.86%)	1 (2.50%)	1 (3.33%)	
N3	0	0	0	
Nx	7 (10%)	0	7 (23.33%)	
**Pathological Stage** ****				0.08
Stage I	57 (81.43%)	29 (72.50%)	28 (93.33%)	
Stage II	9 (12.86%)	8 (20%)	1 (3.33%)	
Stage III	4 (5.71%)	3 (7.50%)	1 (3.33%)	
Stage IV	0	0	0	
**LN Assessment and Upstaging**				
LN assessment *****	63 (90%)	40 (100%)	23 (76.67%)	**<0.01**
Pathologic upstaging	13 (18.57%)	11 (27.50%)	2 (6.67%)	**0.03**
LN upstaging	13 (18.57%)	11 (27.50%)	2 (6.67%)	**0.03**

* Functional tumors: all functional cases were ACTH–producing. ** Smoking: includes current and former smokers. *** Minimally invasive approach: includes VATS and RATS. **** Best available staging—when Nx, N0 was assumed ***** Including lymph node dissection, sampling or unknown type of lymph node assessment **Abbreviations:** ACTH = adrenocorticotropic hormone; CCI = Charlson Comorbidity Index; DIPNECH = diffuse idiopathic pulmonary neuroendocrine cell hyperplasia; FDG = 18F-fluorodeoxyglucose; 68Ga-DOTATATE = gallium-68-DOTA-Tyr3-Thre8-octreotide; FEV1 = forced expiratory volume in one second; F/M = female/male; IQR = interquartile range; LN = lymph node; LOS = length of hospital stay; RATS = robot-assisted thoracic surgery; R0/R1 = negative/positive resection margin; SD = standard deviation; SSA = somatostatin analog; SUVmax = maximum standardized uptake value; VATS = video-assisted thoracoscopic surgery; Tumor location (C/P, % C) = central/peripheral, % central.

**Table 3:** Extent of Lymph Node Assessment by Resection Type.

LN Assessed *	Total (*n* = 70)	Lobar (*n* = 40)	Sublobar (*n* = 30)	*p*-Value
**Hilar LN assessed (N1)** Median (IQR)	2.5 (1–5)	4 (2–6.5)	2 (0–3)	**<0.001**
**Mediastinal LN assessed (N2)** Median (IQR)	1 (0–3)	2 (0–4)	0 (0–1)	**<0.01**
**Total LN assessed**				**<0.001**
0	7 (10%)	0 (0%)	7 (23.3%)	
1–9 LN	42 (60%)	23 (57.5%)	19 (63.3%)	
≥10 LN	13 (18.6%)	12 (30%)	1 (3.3%)	
Not quantified	8 (11.4%)	5 (12.5%)	3 (10%)	
Median (IQR)	4 (2–8.8)	6 (3.5–11.5)	2 (0.5–4)	**<0.001**

* When fragmented nodal tissue was reported, the minimal possible number of LN (one) was assumed. **Abbreviations:** LN = lymph node; IQR = interquartile range.

**Table 4:** Univariate analysis of factors affecting OS and RFS ^1^.

Predictor	OS (*p*-Value)	RFS (*p*-Value)
Clinical variables		
Sex	0.714	0.678
Age	0.266	0.324
CCI	0.907	0.147
Preoperative FEV_1_ (%)	0.775	0.075
Smoking (ever ^2^ vs. never)	0.366	0.420
Previous malignancy	0.458	0.122
Tumor Location (central/peripheral)	0.960	0.575
Clinical tumor size (>2 cm vs. ≤2 cm)	0.351	0.307
Surgical approach (thoracotomy vs. VATS/RATS)	0.816	0.969
Extent of resection (lobar/sub-lobar)	0.940	0.420
PET ^18^F-FDG SUVmax	0.183	0.963
PET ^68^Ga-DOTATATE SUVmax	0.591	0.382
Adjuvant therapy (SSA)	0.459	**0.003**
Functional tumor ^3^	**<0.001**	0.781
**Pathologic features**		
Histologic grade (typical/atypical)	0.548	**0.002**
Necrosis	0.396	**0.001**
Ki-67 index (%)	**0.032**	**0.002**
DIPNECH	0.434	0.861
Lymphovascular invasion	0.336	0.305
Perineural invasion	0.703	0.518
Visceral pleural invasion	**0.006**	**<0.001**
Resection margin status (R0 vs. R1)	0.739	0.712
LN assessment	0.464	0.438
Pathologic upstaging	0.467	**0.049**
Nodal upstaging	0.467	**0.049**

**Abbreviations:** OS—overall survival; RFS—recurrence-free survival; CCI—Charlson Comorbidity Index; FEV_1_—forced expiratory volume in 1 s; VATS—video-assisted thoracoscopic surgery; RATS—robot-assisted thoracic surgery; PET—positron emission tomography; FDG—fluorodeoxyglucose; ^68^Ga-DOTATATE—gallium-68 DOTA-Tyr3-Thre8-octreotide; DIPNECH—diffuse idiopathic pulmonary neuroendocrine cell hyperplasia; LN—lymph node; SSA—somatostatin analog. Charlson Comorbidity Index (CCI). ^1^ Analyses were performed using the Kaplan–Meier method and log-rank test. ^2^ Ever-smokers: includes current and former smokers. ^3^ Functional tumors: All functional cases were adrenocorticotropic hormone (ACTH)-producing.

## Data Availability

All data relevant to this study are available from the corresponding author upon reasonable request.

## Abbreviations

The following abbreviations are used in this manuscript:

AC	atypical carcinoid
ACTH	adrenocorticotropic hormone
DIPNECH	diffuse idiopathic pulmonary neuroendocrine cell hyperplasia
LCNEC	large cell neuroendocrine carcinoma
LNEC(s)	lung neuroendocrine carcinoma(s)
LNET(s)	lung neuroendocrine tumor(s)
LR	lobar resection
NSCLC	non–small cell lung cancer
OS	overall survival
PC(s)	pulmonary carcinoid(s)
PET	positron emission tomography
ProGRPp	plasma progastrin-releasing peptide
RFS	recurrence-free survival
SCLC	small cell lung carcinoma
SLR	sublobar resection
TC	typical carcinoid
